# Five-Direction Occlusion Filling with Five Layer Parallel Two-Stage Pipeline for Stereo Matching with Sub-Pixel Disparity Map Estimation

**DOI:** 10.3390/s22228605

**Published:** 2022-11-08

**Authors:** Yunhao Ma, Xiwei Fang, Xinyu Guan, Ke Li, Lei Chen, Fengwei An

**Affiliations:** School of Microelectronics, Southern University of Science and Technology, Shenzhen 518055, China

**Keywords:** semi-global matching, subpixel interpolation, multi-direction occlusion filling, single precision floating point, disparity refinement, FPGA

## Abstract

Binocular stereoscopic matching is an essential method in computer vision, imitating human binocular technology to obtain distance information. Among plentiful stereo matching algorithms, Semi-Global Matching (SGM) is recognized as one of the most popular vision algorithms due to its relatively low power consumption and high accuracy, resulting in many excellent SGM-based hardware accelerators. However, vision algorithms, including SGM, are still somewhat inaccurate in actual long-range applications. Therefore, this paper proposes a disparity improvement strategy based on subpixel interpolation and disparity optimization post-processing using an area optimization strategy, hardware-friendly divider, split look-up table, and the clock alignment multi-directional disparity occlusion filling, and depth acquisition based on floating-point operations. The hardware architecture based on optimization algorithms is on the Stratix-IV platform. It consumes about 5.6 K LUTs, 12.8 K registers, and 2.5 M bits of on-chip memory. Meanwhile, the non-occlusion error rate of only 4.61% is about 1% better than the state-of-the-art works in the KITTI2015 dataset. The maximum working frequency can reach up to 98.28 MHz for the 640 × 480 resolution video and 128 disparity range with the power dissipation of 1.459 W and 320 frames per second processing speed.

## 1. Introduction

Binocular stereoscopic matching is an essential method in computer vision, [1,2] imitating human binocular technology to obtain distance information. The main goal of binocular stereoscopic matching is to find the corresponding point from two images of the same scene and use the similar triangle principle to generate a reference image disparity map. Based on the disparity diagram generated by binocular stereo matching, a depth map for 3D reconstruction can be generated based on spatial geometric relationships [3,4]. The binocular stereo vision technology has been applied to many fields, including medical diagnosis, unmanned driving [5], virtual reality, three-dimensional reconstruction, robot navigation [6], drone piloting, and virtual reality and augmented reality (VR/AR) applications in need of disparity estimation [7].

### 1.1. Related Works

Among plentiful stereo matching algorithms, Semi-Global Matching (SGM) is recognized as one of the most popular vision algorithms due to its relatively low power consumption and high accuracy, resulting in many excellent SGM-based hardware accelerators. H. Hirschmuller [8] presented stereo processing using SGM and mutual information on accurate applications. P. Dong et al. [9] proposed a coprocessor with a pixel-level pipeline and region-optimized method for semi-global matching in real cases and complimented it on FPGA.

However, vision algorithms, including SGM, are still relatively inaccurate in actual long-range applications. Compared with the algorithm based on deep learning, the traditional binocular stereo-matching algorithm is too inaccurate. Still, it cannot cope with high-complexity scenarios, so its application has been minimal. Specifically, under the condition of occlusion and mismatch, the algorithm generally has an undesirable situation with an increased error rate. These two points are crucial for practical application.

In recent years, stereo matching has still been paid massive attention to instead of disparity refinement, which is significant in obtaining precise depth information. Massive relative works focus on subpixel interpolation. Rui Fan et al. [10] proposed a disparity map with subpixel resolution where a disparity error larger than one pixel may result in a non-neglected difference in the reconstructed road surface.

Besides, Typical disparity filling considers only single row 2-direction filling: the 0-angle and 180-angle filling. Z. Chen et al. [11] offered a post-processing structure of occlusion filling in only 0-angle and 180-angle directions with an error rate of 7.27% using the same database and disparity range. S. Jin et al. [12] presented a left-right check and occlusion filling with a maximum working frequency 93.09 MHz and 11,000 slices, an exceeded resource utilization. Cambium et al. [13] got a disparity whose error rate is 32.9% under KITTI 2015 dataset after occlusion filling with 24.5 K LUTs and 9.1 K registers.

### 1.2. Contributions

This work proposes a disparity improvement strategy with at least 2× smaller FPGA resource usage compared to the previous works. as for the subpixel interpolation, the contribution can be featured in the hardware-friendly divider, split look-up table for cosine function. Furthermore, the disparity optimization post-processing novelty includes the clock alignment multi-directional disparity occlusion filling and depth acquisition based on floating-point operations. Finally, a hardware-friendly architecture is implemented on the FPGA platform with outstanding accuracy and robustness. The hardware architecture based on optimization algorithms is on the Stratix-IV platform. It consumes about 5.6 K LUTs, 12.8 K registers, and 2.5 M bits of on-chip memory. Compared with the state-of-the-art works, the error rate in non-occlusion is reduced by 1% under KITTI 2015 dataset.

### 1.3. Paper Structure

The remains of this paper are organized as follows. We illustrate the proposed subpixel interpolation and disparity optimization post-processing in Section 2. Section 3 elaborates on the implementation of the hardware architecture. Section 4 presents the experimental results with accuracy, hardware-resource usage, and performance. Finally, we conclude in Section 5.

## 2. Algorithm

### 2.1. Framework Overview

**Figure 1:**
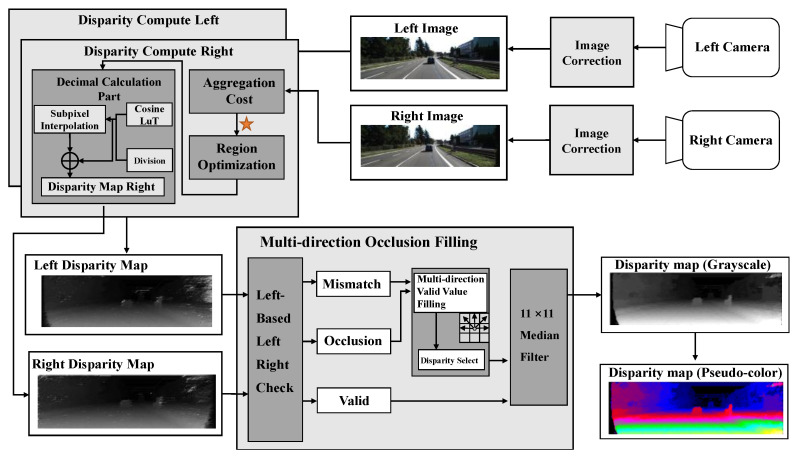
The overall framework of disparity refinement.

The overall flow from cameras to disparity depth map rendering is shown in Figure 1. The main contribution of this work is the post-processing procedure after the aggregation cost (marked with a star) in Figure 1. First, after image corrections and aggregation cost, the proposed sub-pixel interpolation calculates the decimal parts with a cosine look-up table and Newton’s division operator. Further, by carefully conducting a left-right check for the occlusion and mismatching regions, this work processes the left and right disparity values into a multi-direction occlusion filling module for practical disparity values obtained according to different filling rules for occlusion and mismatching regions.

### 2.2. Aggregation Cost with Census Aggregation and Hamming Distance

Zabih et al. [14] proposed a commonly acknowledged census algorithm for calculating matching costs. It can significantly detect the local structural features and achieve high robustness in a light-variable environment. The including census transformation works well in both light and dark conditions. The aimed census flag has been obtained by comparing the gray value of each pixel in the adjacent window to the gray matter of each center pixel of the window. The corresponding explanation Equation containing census transform vector R is shown below:

(1) $$\begin{array}{c} R\left(P\left(i,j\right)\right)=\underset{\left(a,b\right)\epsilon W}{⊗}\xi \left(P\left(i,j\right),P\left(a+i,b+j\right)\right)\\ \xi \left(p,\;p^{\prime }\right)=\{\begin{array}{c} 1,\;p<p^{\prime }\\ 0,\;p\geq p^{\prime } \end{array} \end{array}$$

where ⊗ denotes the concatenation, P(i,j) means the center pixel, and *W* is the matching window. On the condition that the gray value of a certain pixel is greater than the gray value of the center pixel, it is marked as 1. (Otherwise, it is marked as 0.)

**Figure 2:**
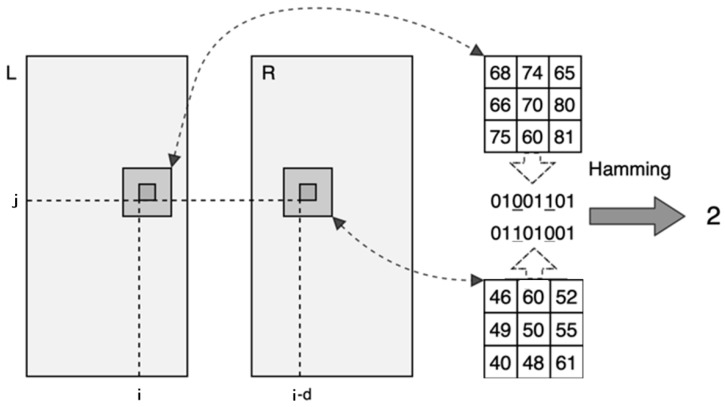
Schematic diagram of the initial matching cost calculation.

The Hamming distance shown in Figure 2 shows that the number of the corresponding bits of two-bit strings is not the same. The calculation method is to perform the XOR operation on the two vectors to obtain a new vector and calculate its number, as shown in Equation (2). The calculated one’s number is the Hamming distance, which represents the initial matching cost of a pixel.

(2) $$C\left(P_{\left(i,\;j\right)},\;d\right)=\sum^{\;}Hamming(R_{R}\left(P_{\left(i-d,\;j\right)}\right),R_{L}\left(P_{\left(i,\;j\right)}\right))$$

In this case, $P_{\left(i,\;j\right)}$ means the position of a pixel in the base image, and $P_{\left(i-d,\;j\right)}$ means the position of the pixel in another image.

**Figure 3:**
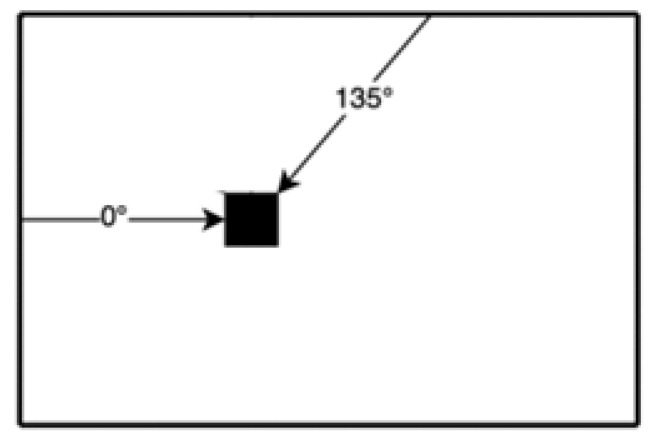
Cost aggregation path.

The Optimization step of the initial matching costs uses a cost aggregation strategy. In the SGM algorithm, a general energy function is established to minimize it for optimization. Further, it considers the one-way dynamic programming method to solve a two-dimensional optimization problem with the most optimized energy function. 0-angle and 135-angle directions resulted in the lowest error rate in experimental results from P. Dong [9], as shown in Figure 3.

### 2.3. Disparity Computing with Subpixel Interpolation Based on Split Cosine Look-Up Table and Practical Divider

Semi-global matching uses the Winner-Take-All (WTA) method to calculate disparity [15]. Each pixel chooses the disparity value related to the minimum aggregate cost as the final disparity. This disparity is usually integer, not adequately reasonable, and desirable in actual cases. The integer is suitable for most circumstances but cannot consider the long-distance situation. According to Equation (7), even if the fraction part’s value is relatively small, the depth can become significant with a low error rate after conducting the multiply. I. Haller and S. Nedevschi [16] proposed an approach of disparity interpolation to solve this problem. Interpolation can increase the decimal place of the disparity, which can effectively improve the accuracy at the line of sight. The difference is declared as the following Equation (3), which consists of two parts. Where d indicates the integer disparity, interpFunction(i) indicates the interpolation value with essential fractions information, and m is the value obtained by the cost aggregation of the pixel.

(3) $$d_{Final}=d+interpFunction\left(i\right)$$

(4) $$leftDif=m_{d-1}-m_{d}$$

(5) $$rightDif=m_{d+1}-m_{d}$$

(6) $$i=\frac{rightDif}{leftDif}$$

(7) $$interpFunction\left(x\right)=0.5-\frac{1}{2}\times cos\left(x\times \frac{\pi }{2}\right)$$

It is important to consider worst-case errors to make the disparity calculation method more robust. Therefore, this work uses the function with the maximum error instead of the sum of errors as the metric for function fitting in Equation (7).

This results in the demand for a hardware-friendly look-up table for the cosine function. Initially containing 360°, this look-up table is divided into six sub-tables, each 64° for better use. Here a projection of processes is formed; for instance, each y corresponds to a function value of a 9-decimal precision cos y, with each projected value effectively expressed through hardware description. Simultaneously, this part introduces the divider with Newton’s method to simplify the calculation process. The representation of the denominator is crucial in most cases. However, it can be replaced as several local changing elements with small intervals typically ranging from one to two, where the parameter can be recorded as binary exponential forms. Finally, the interpolated disparity is obtained.

**Figure 4:**
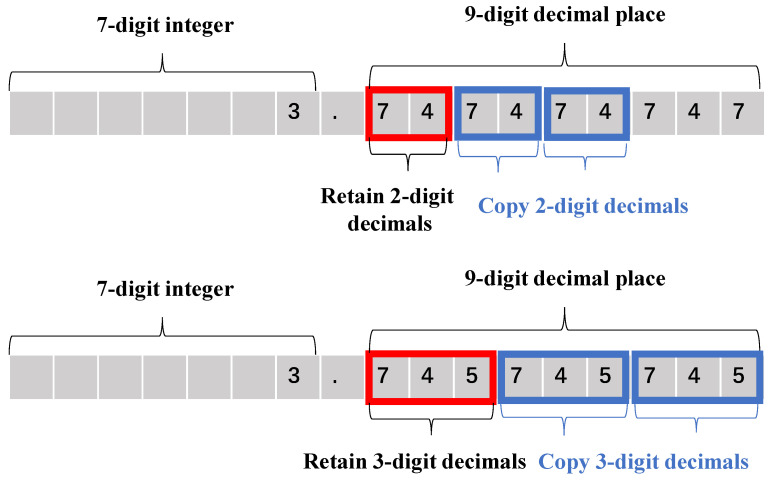
Examples of decimal replication method.

Further, this paper proposes a decimal replication method for the fraction part in interpolation. Figure 4 denotes the instances of the decimal replication method. For example, the result of the calculation may have many decimal places. To reduce the consumption of hardware resources, only two decimal places keep retained after the calculated decimal point, and the remaining decimal places are filled by copying the existing decimal places.

**Figure 5:**
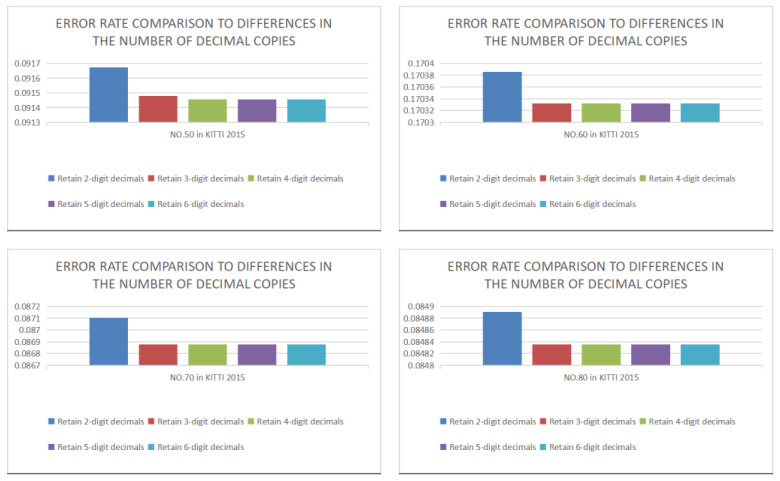
Verification of copied decimal parts.

In Figure 5, the accuracy in retaining only 3-digit decimals is smaller, and the accuracy error with 3-digit decimals or more is almost the same. The accuracy improvement caused by more than three decimal places is tiny but requires more hardware resources. Therefore, 3-digit decimals and replicating them to other decimal places can balance the best accuracy and the minor resource consumption simultaneously.

### 2.4. Multi-Direction Disparity Occlusion Filling with Clock Alignment after Left-Right Check

Disparity filling is the main challenge in disparity refinement due to weak texture conditions, obstacles, etc. The mechanism of left-right detection is as follows: in calculating disparity, the disparity values of left and right maps at the same position are completely different. However, not all differences are unreasonable, and the calculated disparity is reasonable under the threshold. If exceeding, the disparity result is not reasonable. Disparity $D_{L}\left(i,j\right)$ stands for the disparity value of the pixel (*i*, *j*) in the left disparity map whereas $D_{R}\left(i,j\right)$ stands for the value of pixel (*i*, *j*) in the right one.

According to the left image and disparity uniqueness, $D_{R}\left(i,j\right)$ can be obtained by swapping the positions of the left and right images and then gaining the pixel with the same name as an individual pixel in the right image and the disparity value corresponding to the pixel. If the difference between these two disparity values is smaller than one pixel, it can be held. Otherwise, it is abandoned, and [17] proposed to divide it into mismatch or occlusion. The occlusion region is an area of pixels visible on the left view but not on the right view due to foreground occlusion. The occlusion region is more likely in the disparity discontinuity area, where one side is the foreground (larger disparity value) while the other is the background (smaller disparity value). The framework above can be shown in the following Equation (8).

(8) $$\left|D_{L}\left(i,j\right)-D_{R}\left(i-D_{L}\left(i,j\right),j\right)\right|\;\leq 1$$

**Figure 6:**
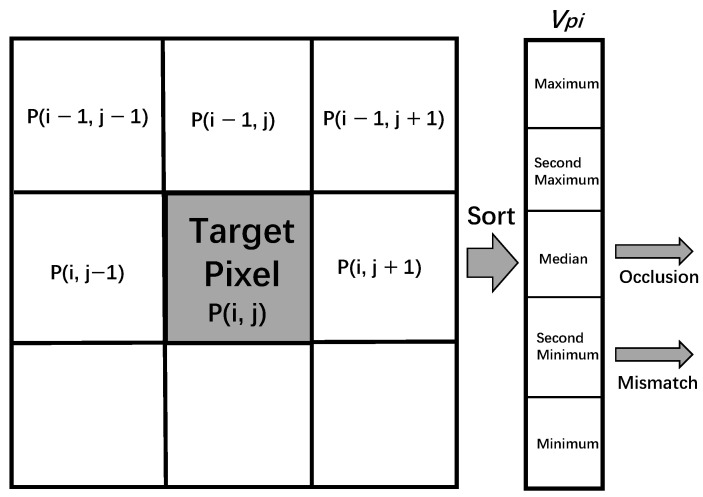
Valid values for occlusion and mismatch.

Generally, the disparity of the surrounding background pixels should be chosen for occlusion conditions when filling occlusions, and the foreground pixels should be avoided [8]. Because the background pixel disparity value is smaller than the foreground, the adjacent minimum is chosen by collecting surrounding valid values. The contiguous pixels of the mismatching region are most likely on the consecutive surface. All pixels in the adjacent area are expected to be concerned when processing occlusion filling. Since the valid disparity values around the mismatching region are relatively equivalent, the middle one is optional. Equation (9) expresses how a resolution-dependent size median filter works for hole-filling. Each hole chooses five valid pixels and then sorts these values from largest to smallest, as shown in Figure 6. If a hole is an occlusion, the second smallest of the five pixels is selected to fill it; if this hole is mismatched, the median of the five pixels is chosen to fill it.

(9) $$D\prime_{p}=\{\begin{array}{c} second\;minimum\;v_{pi\;}\;occlusion\;\\ median\;v_{pi\;}\;mismatch\\ \;D_{p\;}\;other\;cases \end{array}$$

Here, the $v_{pi}$ means the sequence of five-direction valid disparity from minimum to maximum and $D_{p}$ is a defined value set in the beginning.

### 2.5. Floating-Point Operation for Disparity Conversion Depth

Binocular stereo vision fuses the images obtained by the two eyes and observes the differences between them so that we can obtain a distinct sense of depth, establish a correspondence between features, and correspond to the reflection points of the same spatial, physical point in different images. Such a difference, disparity image is defined.

Depth image, also known as distance imagery, refers to an image that takes the distance (depth) value from the image collector to each point in the scene as the pixel value. Thus, to better clarify the depth information of the camera input data, adopting the disparity conversion depth function, Equation (10) is shown below:

(10) $$depth\left(P\left(i,j\right)\right)=\frac{fB}{cZ}$$

where depth(*P*(*i*, *j*)) is the depth of *P*(*i*, *j*), *c* is the pixel size, *B* is the baseline, *f* is the pre-calibrated focus length, and *Z* is the depth of the point from the camera. In this case, we get a more intuitive depth of information instead of a disparity value.

## 3. Hardware Implementation

**Figure 7:**
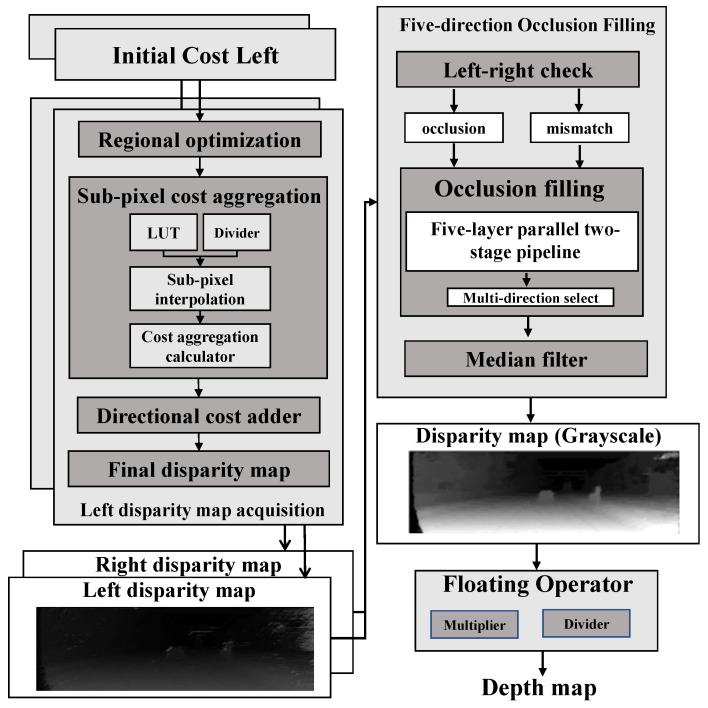
The hardware implementation for sub-pixel cost aggregation and five-direction occlusion filling.

The hardware architecture in Figure 7, emphasizing post-processing disparity optimization, consists of the initial aggregation costs and post-processing. The refined disparity map and the corresponding depth map are expressed in single-precision floating point numbers.

### 3.1. Sub-Pixel Interpolation

**Figure 8:**
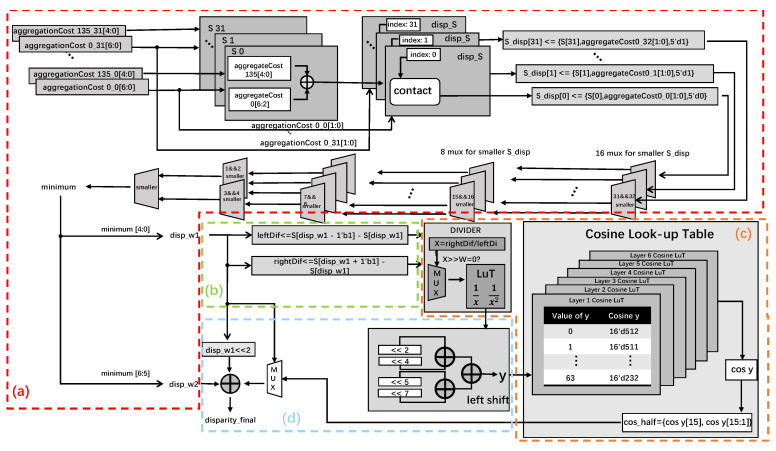
Subpixel interpolation for disparity computation.

**Figure 9:**
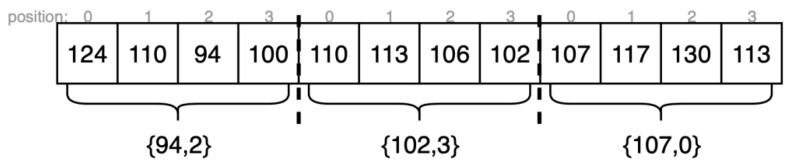
Example of region optimization.

Figure 8 demonstrates the hardware architecture of the sub-pixel interpolation module. (1): enter 32 aggregate values in the two directions, 0°, and 135°. To be able to port on the hardware and ensure accuracy, this work innovatively proposed a processing idea of regional optimization. The 128 initial (usual cases) costs are treated in groups of four, and each group, the smallest generation value is considered the overall generation value of the group. Take Figure 9 as an example. The 12-generation values are divided into three groups. The minimum generation values of each group are found at 94, 102, and 107, and the relative positions of these three generation values in each group are 2, 3, and 0, respectively, so the minimum generation value is spliced with the relative position to obtain three regional optimization values {94, 2}, {102, 3} and {107, 0}, through which the algorithm resource consumption can be greatly reduced. Its advantages are reflected in the process of resource utilization and subsequent cost aggregation.

In Figure 8a, adding aggregated values in all directions can accumulate the matching costs for every pixel. The smallest regional generation value is found through 5 sets of selectors. This minimum region of the minimum optimized cost output is disp_w1, and the position flag output is disp_w2.

As shown in (b) of Figure 8, the difference between the value of the smallest region and the value of the smallest adjacent region is calculated, and the two calculated differences are recorded as leftDif and rightDif, respectively.

In (c) of Figure 8, the result, y, is obtained after division and moving the quotient through the shift operation. Subsequently, using the cosine look-up-table, find out the cosine value corresponding to y, and the result COS_half is obtained by shifting the cosine value right by 1 bit.

Finally, in Figure 8d, the disp_w1 is used to determine whether the region is on edge. If it is not an edge region, multiply the region with the minimum optimized cost by the number of costs in a specific region and add it to the tag flag and the interpolation Equation of intern fuction = 0.5 − COS_half to get the final subpixel disparity value.

### 3.2. Left-Right Check

**Figure 10:**
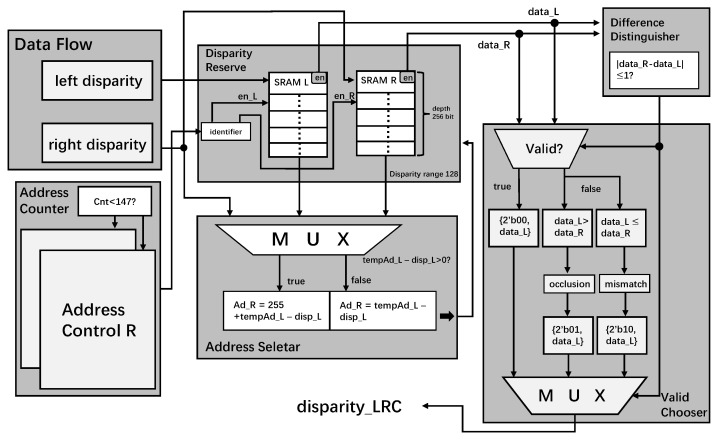
The Left-right Check Architecture.

Figure 10 demonstrates the hardware architecture of the left-right check module. The left-right check module ensures the matching pixels’ effectiveness in accomplishing the filling operation. Based on the inputs and the settlement of the disparity range, 2 SRAMs whose depth is 256 bits are controlled for bit-level reading and writing. The current address of the left disparity and the value are needed to determine the correct address index for the right SRAM to search.

We find the disparity valid whether the absolute difference between left and right disparities is within the threshold range and obtain the output disparity_LRC from the combination of flag bits 2’b00 and the original left disparity. Reversely, on the condition that the difference where the left disparity is numerically bigger than the right one (occlusion) is beyond the threshold, the disparity is invalid, and disparity_LRC is set as a combination of flags bits 2’b10, and the original left disparity. Otherwise, we meet the mismatch condition where the disparity_LRC should be 2’b01 with the original left disparity.

### 3.3. Alignment Multi-Direction Occlusion Filling

**Figure 11:**
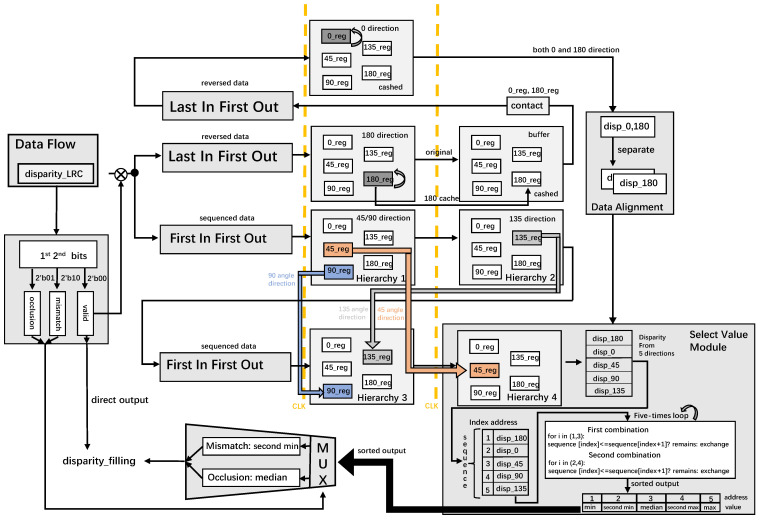
The alignment multi-direction occlusion filling module.

Initially, if the output of the previous module’s left-right check is valid already, the output disparity_filling is set at the same value as the input value. Otherwise, the final disparity value should be gained through adjacent disparity values around the current pixel under different cases. Here we name the process alignment multi-direction occlusion filling described in Figure 11. The first two bits of inputs are cashed to determine different conditions.

**Figure 12:**
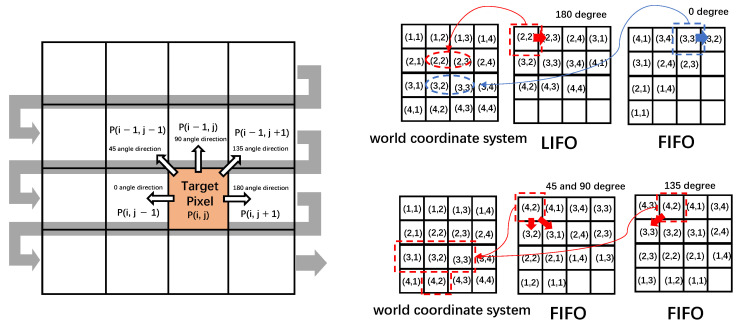
The direct sketch map of multi-direction occlusion filling.

(1) On the condition that the input flag is invalid, the data flow first enters both a LIFO and a FIFO, which hold the same resolution aligned all the time. Then, these pixel coordinates begin to find 180° disparity, 45° disparity, and 90° disparity simultaneously. The value of 180° can be easily gotten through the next pixel coordinate on the condition that the disparity value here is valid. However, the fetch of the 45° and 90° values is relatively tricky. Figure 12 shows a 45° disparity in the northwest direction *P* (*i* − 1, *j* − 1), while the 90° disparity is gained through the absolute above value *P* (*i* − 1, *j*). In the hardware framework, 90° disparity is achieved in the southern direction because of the same length of the FIFO, where the previous pixel will go further in this hardware architecture. Similarly, a 45° disparity is obtained from the southeast direction through this principle.
(2) In the next clock cycle (the second yellow dashed line in Figure 8), on the one hand, the 180° valid disparity value is cashed while the original reversed data flow is retained as well. On the other hand, 135° disparity is obtained through the southwest direction hierarchy.
(3) Disparity values enter another group of FIFO and LIFO, which is used for new line buffers and data initiation, respectively. In hierarchy 3, all registers sustain their values, while a 0° disparity valid value is also found according to adjacent pixels.
(4) Due to the high-standard alignment of the data stream, in the next cycle, five valid disparity values from five directions ranging from 0° to 180° are attained in the select value module. A high parallelism combinatorial logic bubble sort is designed to sort the disparity values in five directions from small to large. Firstly, two adjacent indexes except five values are compared two by two with a result sequence from small to large. Second, two adjacent indexes except index 1 of values are also compared with the result sequence from small to large. After five loops of such logic combination, the final sequenced output disparity value appears, where the median value is selected for occlusion while the second minimum value is selected for mismatch as the final disparity_filling output.
(5) Finally, the output is filtered with a resolution-dependent median filter for better margin information.

### 3.4. Floating Point Operation Process

In this work, only a few meaningful bits of disparity are retained. The other extra bits of disparity are filled with these meaningful bits repeatedly. In this case, there needs only a small number of resources while confirm accuracy.

**Figure 13:**
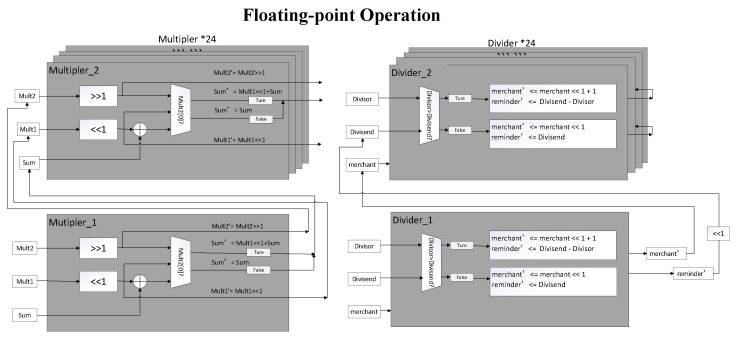
The floating-point operation consists of division and multiplier.

Figure 13 demonstrates the architecture of the floating-point operation process. When performing multiplication and division of single-precision floating point numbers, we propose a special floating point number pipeline multiplier and division device. Based on the IEEE 754 format, the floating-point number of the 32-bit consists of a 1-bit symbol bit (S), an 8-bit exponent bit (E), and a 23-bit mantissa bit (M). When multiplying the floating-point numbers, the exponent bits of the multipliers are added first and multiplied by the mantissa bit (1.M) of the same number of bits. When performing the division operation, the same as the multiplication, the divisor’s exponent is first minus the exponential bit of the divisor, and the decimal number (1.M) of the dividend is divided by the decimal number (1.M) of the divisor. The result float number is represented in the single-precision format after standardizing.

#### 3.4.1. Floating-Point Multiplier

In the design of this paper, the pipeline consists of several cells. A cell is responsible for an addition operation, which outputs the cumulative sum with the shifted multiplier and serve as the next cell’s input. The input for each cell is multiplier 1 and multiplier 2. First, shift multiplier 1 to the left by one bit and multiplier 2 to the right by one bit. If the lowest bit of multiplier 2 is 1, the left-shifted multiplier one is added to the previous cumulative sum; if the lowest bit of multiplier 2 is 0, the cumulative sum does not change. After the execution in this cell, the shifted multiplier 1, 2, and the new cumulative sum are output.

The first cell is first initialized with the original multiplier 1, 2. Since multipliers 1 and 2 have 24-bit, 24 addition operations and 24 cell modules are generated. The product of the outputs is a fixed 48-bit in general, such as 10.M_3_, 01.M_3_, 11.M_3_.

Under the IEEE 754 standard, the leading number of the significand is always 1. Consequently, a leading one can be implied, and the explicitly represented part of the significand lies between 0 and 1. After normalization and rounding to the nearest value, the 48-bit product represents in the standard format, e.g., 24-bit 1.M.

#### 3.4.2. Floating Point Divider

Similarly to the multiplier above, a cell is responsible for a single subtraction operation and shift operation. If the divisor is less than or equal to the dividend, the dividend is minus the divisor, and the quotient shifts left one place and added 1 to the lowest bit. If the divisor exceeds the dividend, the quotient shifts left one place, and the dividend becomes the remainder. The remainder of the last cell outputs and shifts left one place, then serves as the input dividend of the next cell.

The first cell is initialized with the original divisor and dividend. Since both the divisor and the divisor are 24-bit, 24 subtraction operations will be performed, and 24 cell modules will be generated. The final quotient is fixed 24-bit in general, either 1.M_3_ or 0.M_3_. Furthermore, the quotient will be represented in the standard IEEE 754 format, e.g., 1.M, like the product’s rounding and normalization.

## 4. Results and Discussion

The proposed algorithm is implemented on Intel Stratix IV (EP4SGX230) and Stratix V (5SGXEA7N2F45C2N) FPGA in Verilog HDL. We leverage the simulation results on the KITTI 2015 data sets to validate the accuracy. The hardware resource usage and power dissipation can be found in the synthesis results from the FPGA EDA tool, namely Quartus Prime. In this work, the disparity range is set to 128 because it is a typical value in the previous works for both VGA and XGA video input. Besides, we take VGA resolution for the fare comparison with the convincing hardware resources in [12,13].

**Figure 14:**
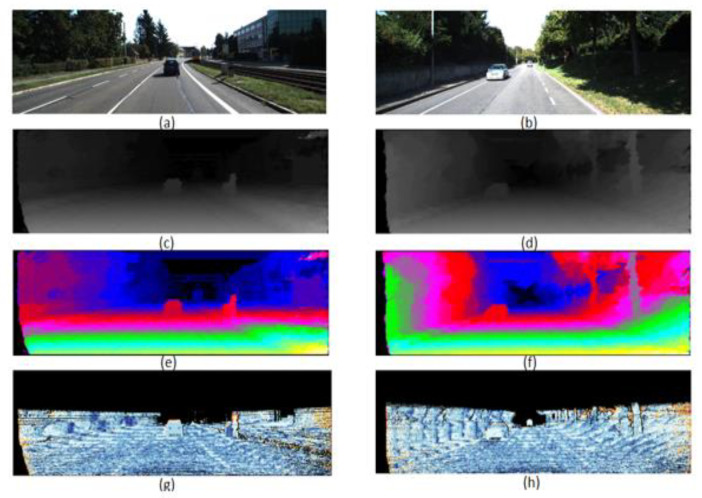
(**a**) No.110; (**b**)No.128; (**c**) Grayscale disparity of (**a**); (**d**) Grayscale disparity of (**b**); (**e**) Pseudo-color disparity of (**a**); (**f**) Pseudo-color disparity of (**b**); (**g**) Error of (**a**) (noc:2.77%, occ:4.48%); (**h**) Error of (**b**) (noc:3.80%, occ:6.06%).

**Figure 15:**
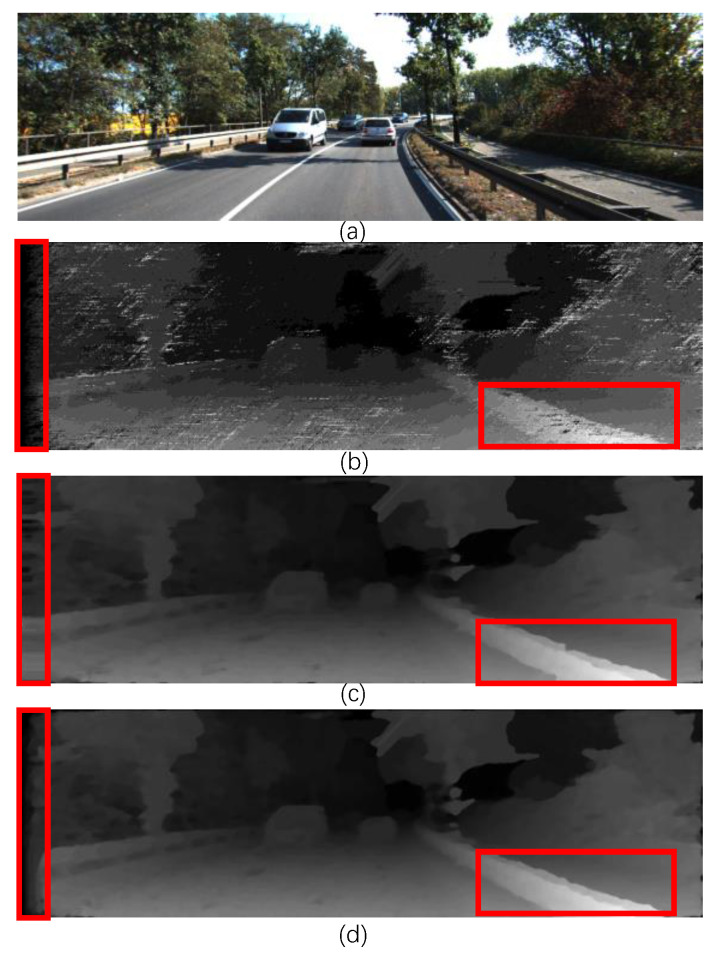
Disparity map comparison of (**a**) original No.174 image in KITTI 2015; (**b**)The disparity map without post-processing; (**c**) use only 0 and 180 angle direction filling with error rate 7.27%; (**d**) use proposed five-direction filling with error rate 6.04%.

The simulation results in Figure 14 show the gray disparity map, pseudo-color disparity map, and error map of NO.110 and NO.128 images in KITTI 2015. Figure 15 shows the proposed gray disparity map of NO.174 image in KITTI 2015 compared with only 0 and 180-angle directions filling with the original disparity map without post-processing. From error rates of 7.27% in two-direction single-row filling and 6.04% in presented five-direction spanning multiple rows, the filling efficacy increases, especially in the red box. The quality of the disparity map is enhanced through five-direction spanning multiple rows, where the boundaries are more refined and less affected by the surroundings.

In Table 1, considering this work aims at disparity refinement, we only compare the hardware resources of the post-processing module in previous works since this part of the contribution is relatively scarce in the current study.

In Table 2, these compared works propose an entire system where the inputs are camera streams and outputs are disparity videos. Unlike them, this work contributes to disparity refinement; therefore, we only compare the output error rate of the proposed work with plentiful advanced works from journals and conferences under non-occlusion and occlusion situations. We take the 20 best error rates of images from the KITTI 2015 dataset and obtain their average. Generally, this work has achieved an accuracy of 4.61% in the non-occlusion situation and 6.04% in the occlusion situation. The presented work offers more precise results than the experimental measurements in [9,18,19] for non-occlusion conditions, whose error rates are 6.54%, 5.66%, and 6.58%, respectively. Meanwhile, for occlusion consideration, our work has a lower error rate than the error rate of 7.52% from [20], 6.88% from [21], and 7.27% from [9], but performs worse than [19]. This work reduces the error rate of 2% in non-occlusion and 1% in occlusion conditions.

**Figure 16:**
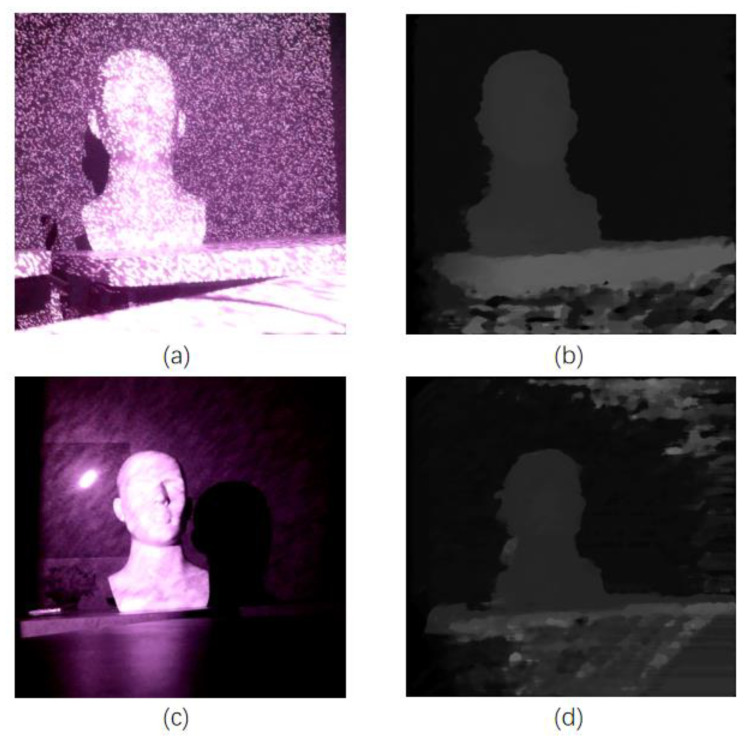
(**a**–**c**) image of real world obtained by camera; (**b**) Real time output disparity map of (**a**); (**d**) Real time output disparity map of (**c**).

**Figure 17:**
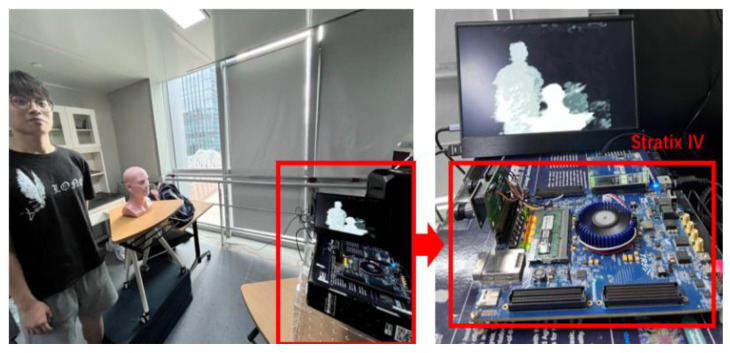
FPGA demonstration.

Meanwhile, this work aims at stereo vision post-processing with high-precision infrared speckle structured light in future work. The infrared camera ignores dark backgrounds and weakly textured backgrounds, providing a more accurate match to the original image. The real-world infrared speckles stereo image and obtained disparity map are shown in Figure 16. Figure 17 shows that the five-direction occlusion filling method proposed in this work has improved the edges of the image by FPGA implementation with MT9V034 global-shutter CMOS Image Sensors.

The hardware architecture based on our optimization algorithms is completed and implemented on the Stratix-IV platform, and it consumes about 5.6 K LUTs, 12.8 K registers, and 2.5 M bits of on-chip memory. The maximum working frequency can reach up to 98.28 MHz for the 640 × 480 resolution and 128 disparity range with a power dissipation of 1.459 W and a processing speed of 320 frames per Second. Compared with work [12] and [13], although our post-processing module consumes more than 4000 registers, it only uses less than 1/10 LUTs of their work and presents high disparity accuracy. Meanwhile, the memory usage in our work has been reduced to less than about 1/9 in [13] and even more than [12]. In addition, Stereo MATLAB Calibration Toolbox is used in the proposed hardware solution, meaning that it is a very general solution to be adopted after calculating the calibration parameters with the software tool most widely used for camera calibration.

## 5. Conclusions

In conclusion, this paper proposes a high-accuracy hardware-friendly architecture for post-processing a typical stereo vision Semi-Global Matching algorithm to improve disparity precession on FPGA platforms through subpixel interpolation and five-direction occlusion filling, after which the depth information is obtained as well through a floating-point operator. The proposal aims to enhance the accuracy of the disparity map for real cases.

The overall purpose of this disparity refinement processing is concluded as the following. (1) Initially, this paper proposes an interpolation algorithm obtaining a fractional disparity value with subpixel information instead of an integer pixel accuracy by adding a divided cosine look-up table and Newton’s division operator. Accuracy is massively promoted by adding these essential fraction parts and copying the three decimal places after the decimal point according to the best precision. (2) Secondly, the left-right check module is utilized to correct the effects of both left and right disparity maps. (3) Then, to obtain an optimized disparity map with relatively low hardware resources, a five-direction occlusion filling considering pixel coordinates around the center is presented with different real situations. Based on that, according to the overlapping bubble ordering framework, we propose a parallel combinatorial logic that can efficiently get the arrangement permutations from small to large in those five directions simultaneously. (4) Consequently, the final disparity value is converted into more direct depth information for real sense by using a single precession floating operation, including floating-point multiplier and division.

This work has been verified on the FPGA platforms and compared with several advancing kinds of research, which are adequately hardware-friendly and convincing with the error rate of the output disparity map of nearly 4.61%, apparently superior to other work.

The deep neural network (DNN) often produces good depth estimation in the literature [22,23], while the hardware usage of an accelerator is incompatible with the requirement of real-time and low power. In the future, we may take advantage of the DNN method only for disparity refinement.

## Figures and Tables

**Table 1:** Comparison of FPGA resources (-: not mentioned).

	This Work		[12]	[13]
Resolution	**640 × 480**	640 × 480	640 × 480	640 × 480
Disparity Range	**128**	128	64	128
FPGA Platform	**Stratix-IV**	Stratix-V	Xilinx XST J.33.	Stratix-IV
LUTs	**5.6 K**	5.76 K	60 K	12.6 K
Registers	**12.8 K**	12.9 K	-	9.1 K
On-Chip Memories (bits)	**2.5 M**	2.5 M	2.06 M	2.8 M
Frame per Second (fps)	**320**	375	302	-
Frequency (MHz)	**98.28**	115.3	93	-
Power Dissipation (W)	**1.459**	0.876	-	-

**Table 2:** Comparison of the error rate.

Work	KITTI2015	
	Noc (Error Rate)	Occ (Error Rate)
JSSC 2019 [20]	-	7.52%
TCSVT 2019 [21]	-	6.88%
TCSVT 2021 [18]	6.54%	7.44%
TCSVT 2021 [19]	5.66%	5.84%
TCAS-I 2021 [9]	6.58%	7.27%
This work	4.61%	6.04%

## Data Availability

Not applicable.
